# Positive Clinical Outcomes Are Synergistic With Positive Educational Outcomes When Using Telehealth Consulting in General Practice: A Mixed-Methods Study

**DOI:** 10.2196/jmir.4510

**Published:** 2016-02-08

**Authors:** Patricia Knight, Andrew Bonney, Grigorijs Teuss, Michelle Guppy, Danielle Lafferre, Judy Mullan, Stephen Barnett

**Affiliations:** ^1^Graduate School of MedicineUniversity of WollongongWollongongAustralia; ^2^Discipline of General PracticeSchool of Rural MedicineUniversity of New EnglandArmidaleAustralia

**Keywords:** telehealth, medical education, patient benefits

## Abstract

**Background:**

The use of telehealth technology to enable real-time consultations between patients and specialist services (to whom travel may be an impediment to the patient’s care) has recently been encouraged in Australia through financial incentives. However, the uptake has been both fragmented and inconsistent. The potential benefits for patients include access to a broader range of specialist referral services, cost and time saving, and more rapid access to specialist services and a continuum of care through the triangulation of interaction between patient, primary health care providers (general practitioners and nurses), and specialists. Enhanced broadband connectivity and higher-grade encryption present an opportunity to trial the use of telehealth consulting as an intrinsic element of medical education for both medical students and doctors-in-training within rural practices and Aboriginal Medical Services.

**Objective:**

This paper discusses the reported, and varied, benefits of telehealth consulting arising from a multisite trial in New South Wales, Australia. The purpose of this study is to encourage the use of selected telehealth consultations between patients in a primary care setting with a specialist service as an integral aspect of medical education.

**Methods:**

The trial closely followed the protocol developed for this complex and multiaspect intervention. This paper discuses one aspect of the research protocol—using telehealth consultations for medical education—in detail.

**Results:**

Qualitative and quantitative analyses were conducted. In the quantitative analysis, free-text comments were made on aspects of Telehealth Consulting for the patient, concerning the quality of the interactions, and the time and cost saving, and also on the leaning opportunities. Students commented that their involvement enhanced their learning. All respondents agreed or strongly agreed that that the interpersonal aspects were satisfactory, with some brief comments supporting their views. In the analysis of the qualitative data, five themes emerged from the analyses concerning the educational benefits of Telehealth Consulting for different levels of learners, while three themes were identified concerning clinical benefits.

**Conclusions:**

The results demonstrated strong synergies between the learning derived from the telehealth consulting and the clinical benefits to the patient and clinicians involved.

## Introduction

The use of telehealth technology to enable direct, real-time consultations between patients and specialist services located at a distance from each other has recently been encouraged in Australia through financial incentives [1]. Although the uptake has been fragmented and inconsistent [2,3], potential benefits for patients are in a range of enhancements across the continuum of their care. These include improved access to a broader range of specialist services, cost and time saving, more rapid access to specialist services, and a better continuum of care through the triangulation of interaction between patient, primary health care providers (general practitioners [GPs] and nurses), and specialists, who may be based distally from the patient, often in metropolitan areas [4]. A recent Australian study demonstrated that telehealth consulting reduced patients’ traveling time by a mean of 50 minutes (from median 80 mins to 30 mins) [5]. However, travel times for those living in rural or remote Australia may be considerably greater than this.

The practice of telehealth consultations, supported by health care staff (doctors, nurses, and allied health workers) in general practice, has been incentivized in the last 4 years in Australia, which has led to an increase in usage rates [1]. However, the rate of uptake has been fragmented for multiple reasons with the current rate being only 0.24% of all consultations conducted via telehealth consultations [1]. Medicare rebate records indicate there are 6000 specialist telehealth consultations per month in Australia [1] .The incentives were provided for telehealth consultations between patients and specialists through GPs, nurses, Aboriginal health workers, and aged care facilities, and they were limited to nonmetropolitan areas (with exceptions for Aboriginal Health Services and aged care facilities).

Technological advances, resulting in enhanced broadband connectivity and higher-grade encryption, presented the opportunity to trial the use of telehealth consulting from general practices to remote or distant specialist services as an intrinsic element of medical education. This was for medical students on longitudinal rural placements, doctors-in-training primarily in rural practices, and places where supportive Medicare payments were available for Aboriginal Medical Services. There is a paucity of data describing potential educational benefits of telehealth consulting for those working within or on placement in primary care.

Because telehealth consulting is a dynamic and developing aspect of patient care, it is imperative that potential users are aware of current and evolving technological developments to potentially incorporate them into regular practice. This is particularly relevant to new applications, which may benefit patient care, specialist accessibility, and medical education where service provision may be difficult to access.

This paper discusses the reported benefits, both educational and to patient, of telehealth consulting from a multisite trail in New South Wales, Australia. This trial was funded through the Federal Department of Education and Training’s Broadband Enabled Education Skills and Services Programme, which ran from 2013 until December 2014. The project was led by the University of Wollongong team working in a collaboration with several other medical schools (University of New England, University of Newcastle, Deakin University, and University of Notre Dame, Sydney) and 2 vocational training organizations (Coast City Country General Practice Training and GP Synergy).

The trial closely followed the protocol developed for this complex and multiaspect intervention [6]. This paper discusses the results, in terms of reported benefits, that have been derived from real-life patient telehealth consultations. It is limited to this aspect of the research.

It is important to define telehealth consultations in this context because this term has evolved from the earlier term “telemedicine” [7]. The International Organization of Standardization defines *telehealth* as “the use of telecommunication techniques for the purpose of providing telemedicine, medical education, and health education over a distance,” which encapsulates the objectives of this study [3].

In the health professions, learning by use of telehealth consultations, although an emerging issue, has had limited research. A systematic review revealed that there were few randomized controlled trials comparing traditional face-to-face learning with telehealth learning [8]. However, the authors of the systematic review concluded that the use of telelearning does enable equivalent opportunities for learning compared to face-to-face within medical education, particularly for rurally based services. There are advantages for rural doctors in using telehealth, through both reduction in isolation and enabling peer communications [9]. This can facilitate retention of rural doctors by reducing their perceptions of isolation from peers and face-to-face learning opportunities [4]. In spite of these advantages, a study from the United Kingdom reported concerns about the introduction of new technology, how patients would cope with using it, and of perceived changed standards of care associated with the use of e-technology [10].

### Can Telehealth Consultations Contribute to Medical Learning?

Based on adult learning theory, the University of Wollongong has a longitudinal integrated placement or clerkship for all their postgraduate medical students after 2.5 years of their 4-year degree course. The rural integrated clerkships are based on a model pioneered and extensively evaluated in Australia by Flinders University [11]. The evaluations have demonstrated that long-term integrated clerkships excel in exposure to patient experiences and interdisciplinary working (ie, experiential learning through learners working alongside and with colleagues across a range of health care disciplines), but may lack exposure to specialists [11]. The majority of University of Wollongong students are placed in rural areas and their involvement within this unfamiliar community where their 38-week placement occurs is encouraged both professionally and socially. The students spend 2 days working in general practices, primarily consulting with patients in parallel with and under the guidance of their supervising doctors [12]. Students have access to undifferentiated patients and learn through the skills, expertise, and teaching of their GP preceptors, and from the patients themselves [12]. Additionally, the students spend time in hospitals and with community-based services within the area of their placement.

The purpose of this study was to encourage the use of selected telehealth consultations between patients in a primary care setting with a specialist service for medical education and to evaluate the clinical and educational outcomes of this experiential learning by a medical student or doctor-in-training involved in the live telehealth consultation.

### Ethics Approval

Ethics approval for the research was obtained through the University of Wollongong’s Human Research Ethics Committee (reference HE13/238).

## Methods

The study commenced in late 2013 and concluded in December 2014. A total of 10 medical practices were recruited across 2 study groups based at the University of Wollongong and the University of New England in Armidale. Nine were from rural areas and the other was an urban-based Aboriginal medical center’s 2 practices. A series of telehealth consultations, from general practices to a distant medical specialist, were evaluated. For these, the patient and specialist consented to the student or doctor-in-training’s involvement and participation in the consultation.

Practice staff and clinicians from practices, which had little or no previous experience with telehealth consultations, completed a skills training module on implementing telehealth consultations in early 2014. Additionally, all University of Wollongong students completed a similar training module before starting their integrated placements. The training included 2 cohorts of students. The first completed the module in May 2013 (n=74) and the second cohort in July 2014 (n=76).

Two methods of data collection were employed. Quantitative data (the first data collection method) were derived from written evaluations of telehealth consultations. The written evaluations contained 5 questions, with a Likert scale answer, and also invited open comments. There were questions on the educational and clinical benefits, the technical quality, and the interpersonal aspects of the consultation. These, along with the appropriate consent forms, were returned to the research team and analyzed at the conclusion of the trial.

The second data collection method included qualitative data, which were obtained from semistructured interviews conducted with GPs, practice managers, nurses, patients, and learners (students or doctors-in-training) associated with each of the participating practices. The interviews were conducted at the 10 participating medical practices by different members of the research team. Interviews were conducted at baseline, when the practice joined the research, during the trial, and at the end of the trial. Student interviews were conducted for the first cohort of students at the end of their placement and for the second cohort at the start of their placement and at the end of 2014. The interviews from each practice were grouped to form a multisite case study. Five practices were selected for analysis of this aspect of the study, aiming for a range of practice locations and distances from the major metropolitan center of Sydney. Characteristics of these practices are described in Table 1 and a description of the structure of Rural, Remote and Metropolitan Areas (RRMA) classification are presented in Table 2.

**Table 1:** Characteristics of the 5 case study sites.

Site	Rural area	Distance from Sydney (km)	Total interviews, n	Interviewees
Site 1	RA2	617	14	GPs, practice manager, nurse manager, mental health nurses, practice nurses, students
Site 2	RA2	112	7	GPs, practice manager, Prevocational General Practice Placement Program (PGPPP), students
Site 3	M2; Aboriginal Medical Services, Urban	88	7	GPs, practice manager, practice nurses student
Site 4	RA2	219	6	GPs, practice manager, students
Site 9	RA2	476	7	GPs, practice manager, PGPPP

**Table 2:** Structure of the Rural, Remote and Metropolitan Areas (RRMA) classification.

Zone		Category
**Metropolitan zone**		
	M1	Capital cities
	M2	Other metropolitan centers (urban center population >100,000)
**Rural zone**		
	R1	Large rural centers (urban center population 25,000-99,999)
	R2	Small rural centers (urban center population 10,000-24,999)
	R3	Other rural areas (urban center population <10,000)
**Remote zone**		
	Rem1	Remote centers (urban center population >4999)
	Rem2	Other remote areas (urban center population <5000)

Within the participating practices, telehealth consultations were selected by the GP preceptor to be suitable as potential learning experiences.

The interviews were conducted by 4 experienced members of the research team (PKB, GT, DL, and JB). Participant selection was purposeful, selecting those within the practice who were involved in the project’s implementation. A total of 81interviews were conducted, face-to-face, in the participating practices. There were no refusals to participate. Because the participants were interviewed at the beginning, during, and after the trial period, reference was made to individual perspectives to establish if changes had occurred.

All interviews were recorded and transcribed verbatim by an independent transcriber and checked for accuracy by a member of the research team. After a review of all data collected, the analytical approach was based on case studies, which consisted of all the interview data from a particular participating practice analyzed as a case study site.

At the completion of analysis of all interviews from the 5 selected practices, it was considered that data saturation had been met and no “new” or novel themes were emerging from the data. Three coders worked independently on the data, with at least 2 researchers independently coding each case study (ie, all interviews associated with one particular general practice). Based on the grounded theory approach, emergent themes were tabulated by the 3 researchers and discussed to confirm concurrence of perception [13].These emergent themes were then confirmed by other members of the research team. The rigor was compared with the standards expressed as consolidated reporting criteria qualitative research [13], which covers the range of criteria, including study context, findings, methods, and research team.

This dual evaluation process, using qualitative and quantitative methodologies, enabled a mixed-methods presentation of the findings.

## Results

### Quantitative

The quantitative analysis questions and responses are presented in Table 3. There was additional space for comments.

**Table 3:** Quantitative evaluation results of telehealth consultations from participating medical practices (total evaluations completed: n=38).

Question: Thinking about the telehealth consultation in which you were just involved, to what extent do you agree with the following statements?	Response, n (%)				
	Strongly disagree	Disagree	Neither	Agree	Strongly agree
I think the technological aspects of the telehealth consultation (image, sound quality, or reliability) were satisfactory for its purpose.			2 (5)	17 (45)	19 (50)
I think the clinical aspects of the telehealth consultation (history taking, examination, or discussion of management plan by video consultation) were satisfactory for its purpose.		2 (5)	1 (3)	23 (60)	12 (32)
I think the interpersonal aspects of the telehealth consultation (interaction between doctors and patient via video consultation) were satisfactory for its purpose.				23 (60)	15 (40)
I think the telehealth consultation was valuable as a student/registrar learning experience.		1 (3)	2 (5)	21 (55)	14 (37)

There were free-text comments made on aspects of telehealth consulting for the patient, concerning the quality of the interactions, the time and cost savings, and also on the leaning opportunities. Students commented that their involvement enhanced their learning.

One GP commented, on his first involvement in telehealth consulting:

Excellent. Screen “melted away” [and was] no barrier

GP, site 2

The technical aspects were rated as “neither satisfactory or not satisfactory” (ie, neutral) by one student due to poor picture quality and one GP due to sound quality issues, which also impacted patients with hearing difficulties. For the 3 responses that were either neutral or not satisfactory, 2 were related to the same consultation and the student and GP both felt the examination was difficult; however the student described it as “good rapport, in a short consultation, and to be an effective use of time” and a similar comment was made by the other student whose experience was neutral.

All respondents agreed or strongly agreed that that the interpersonal aspects were satisfactory, with some brief comments supporting their views.

Three evaluations, all by students, rated their experiences in terms of learning as either “not valuable” (n=1) or “neither valuable nor not valuable” (n=2). Their comments demonstrated that the consistent issue with the 3 consultations that were considered less valuable was the shortness of the consultation.

### Qualitative

In the analysis of the qualitative data, 5 themes emerged from the analyses concerning the educational benefits of telehealth consulting for different levels of learners:

1. Investment and support
2. Patients as educators
3. Evolving real patient learning
4. Mental health learning
5. Job readiness

Three themes were identified concerning clinical benefits:

1. Continuity of care
2. Timeliness
3. Normalization

### Administrative Investment Within the Practices and Practice-Wide Support Enhances Educational Outcomes

Having a designated telehealth “organizer” was seen as a worthwhile investment in practices. An administrator with responsibility and appropriate support to organize telehealth consultations was frequently seen as the foundation for efficient implementation of telehealth consults and the effective use of telehealth consulting as an educational tool:

I have a referral, go and ask the telehealth person, give it to them they’ll sort it all out, and that has actually been great but there are just lots of little steps in that in terms of knowing who to book, how to book, how to set it all up, how to set up the tech on both sides and then set up the bookings...particularly in terms of this trial to make it a teaching experience.

GP, site 2

The support resulted in mutually beneficial outcomes with unanticipated benefits from the practice staff and students working together:

When it comes to the appropriateness of having students in with the telehealth consultation we find that it’s well received on a couple of levels, one because we can provide the students with an opportunity to listen and take in the information and absorb. Two, they are there to support the clinicians and nurses in the treatment of the patient and the patients seem to enjoy it because it involves them more...it creates that relationship that’s required to really get patients to open up.

practice manager, site 1

Practice-wide support was viewed as a facilitator for clinical and educational benefits, with principals referring to members of staff and their roles, and to “we” being interested in setting it up, with the decision being supported by all those working in the practice

The nurses are very good at supporting telehealth consults now, realistically the nurses…are doing a lot of the tech support for the telehealth and they have been the ones who have been setting up the telehealth consults with the specialist and a little bit the health worker have been supporting but it’s basically been [practice manager] and [practice nurse] who have been doing the set up for the telehealth consults with the specialists for the patients.

GP Site 1

I guess we were interested in the perspective of setting it up for ourselves clinically but also that we have the Phase 3 students and using it for both students and registrars as a way of teaching.

GP, site 3

### The Patient’s Role in Medical Education and the Educational Opportunities for Family and Others

Through a wide range of cases, including psychiatry, rheumatology, and pediatrics, the patients enjoyed having what they perceived to be a valuable role in the medical education of the student and other learners:

The patient was very happy for everybody to be involved and in fact he was quite open and seemed to almost enjoy having this to be part of a teaching experience.

GP site 2

The direct observation of consultations, we find patients really enjoy that; having the students and knowing they are being part of educating the future generation of health professionals.

practice manager, site 1

### Evolving a New Form of Parallel Consulting Through Engagement of a Specialist in Patient and Learner Education

The participants in telehealth consulting were varied. The majority of the consultations, which all were between the patient and a distal specialist, were with the GP and student or doctor-in-training, and sometimes with the practice nurse with the learner. Although the learning opportunity was primarily for the medical student, the GPs also reported other benefits for those who became involved in the consultation, through being able to be a part of a parallel consulting experience involving the GP and patient, the specialist and patient, and the GP and specialist:

Having that access to a consultant is great...Often you might have seen that same patient with the GP. So to see the difference in how they approach the same condition from the GP to the consultant, that was nice to see as well.”

student 2, site 1

One GP saw a possible dual role for the doctor-in-training involving a different aspect of parallel consulting with the specialist, a role that the student might not have been suited to:

When the student is there they really are obviously there for a teaching experience and the experience they’ve had with patients so far they’re very happy with that. From a practical point of view the registrar can actually add some clinical value just in terms of their seniority of experience...because the registrar had actually done it, they were able to finish the consultation, organize the script, book the next appointment in and do a hand over to me and bill to Medicare, whereas a student obviously couldn’t have done any of those things without me being there so that’s probably a practical role.

GP, site 2

Doubts concerning telehealth consultations were alleviated for some with the development of new forms of learner participation resulting:

I was just surprised with how well it flowed and the Internet line was great, it was very easy to communicate, there were no interruptions into the Skype. I think as well I was surprised how well the patient was able to interact with the clinician...it ran exactly how we had prepped for them in the phase 3 intro. [Clinical skills training at University of Wollongong] So he introduced himself and then got the patient to introduce himself and said, look, who else in the room? So I introduced myself and the clinician actually involved me throughout...The rapport throughout the consult was very, very good.

student, site 1

New forms of learner participation extended to the GPs’ involvement in the telehealth consultations as well:

Well I think it offers a few things. We almost never get the opportunity to sit in when your patient goes to see the specialist so it’s a unique situation of being able to do that. One of, I think, our greatest educational tools as a GP and particularly in the past, was specialist letters but better still, to actually be there and be able to say “No I don’t...so what’s that bit” and those sorts of things so I think that’s a fabulous part of it.

GP, site 9

### Educational Opportunities Specific to Mental Health Consulting

The majority of telehealth consultations in the participating practices were with psychiatrists. Many psychiatric consultations were considered by the referring GP to be inappropriate for learners to be a part of. However, those that did involve a learner were well received by patient, GP, specialists, and learners alike. They facilitated a breadth of learning opportunities and insights into the unique aspects of psychiatric consultations to which the learners and GPs felt they had not previously had access:

My perception of the trial at the start was that it was really for the students, and that it was mainly pragmatic education about how do you tele-psychiatry because in your lifetime you are going end up doing more of it and with [National Broadband Network] NBN rollout in regional areas it’s just going to be a more of a fact of life. But I think the unexpected side effect for us anyway that it was an educational experience in a clinical context so it ended up learning how to do tele-Consulting but also ended up being a psychiatry modeling training experience for multi levels of learner.

GP, site 2

It’s great with mental health patients which is one of the things I was skeptical about...I haven’t had any patients that have been negative about it. I know that they discuss pretty much anything and everything the way they would on a one-on-one basis in person.

nurse manager, site 1

So, the opportunity to be in consultations which are usually quite guarded and where patients usually don’t like for a third party to be present and especially the history taking skills and how to build rapport with patients with mental illnesses...It’s really opened my eyes on how history taking can be done differently to glean more information...I have to say prior to this learning experience I probably treated mental health consultation very similarly to any other health consultation.

Prevocational General Practice Placement Program [PGPPP] doctor-in-training, site 2

Mutually beneficial [to patient and student] too because I learnt a lot...Also it was private practice mental health, which we haven’t been exposed to before in our psych rotation since inpatient and it’s a completely different patient population to private practice...So that was great actually. It gave psych a different light.

student, site 1

...to see somebody [the specialist] who was able to establish rapport and provide a lot of emotional support, some diagnostic input and then a whole lot of safety netting and then arrange the follow-up consultation, such that the patient said that it was probably the best psychiatric experience he had ever had in terms of support.

GP, site 2

### Enhancement of Job Readiness

The majority of the research was in rural general practices. General practice trial participants indicated that learners demonstrated improved job readiness by being exposed to consultations with specialists and the use of telehealth consulting as an integral aspect of patient care:

It’s really useful and as a registrar, it’s a good learning tool for me. I’d love to, you know, continue doing the telehealth if I have a chance. I’ve never done it on my own but if I have a patient who will benefit from the telehealth I would definitely do that.

doctor-in-training, site 9

Job readiness enhancement was also seen in multidisciplinary team work exposure as a result of telehealth consulting:

It [telehealth consulting] gives a unique insight for training...getting a comprehensive understanding of what a real and true multidisciplinary care model is for a patient. You get some real insight what other Allied Health professions and disciplines are doing with patients...seeing the sort of questions that are being asked and the types of topics that are being focused on

practice manager, site 1

Augmenting skills through working with experienced health care workers in Aboriginal health and enhancement of cultural awareness linked with being involved in the telehealth consultations were other work readiness advantages:

The improved quality with the terminology and linguistic barriers and all that sort of area I think having a medical student observing that will improve their way—given when they become a GP or an Allied Health Service provider of how to communicate with the Aboriginal, Torres Strait Island community because they will observe the challenging questions that the practice nurses had to bring down to the grass roots level in terminology exchange and they will take that on board with them and that will make them become culturally safe practitioners.

practice manager, site 3

Related to work readiness was the perception that role of telehealth consulting in reducing rural isolation for health care staff in a rural situation:

I think one of the reasons—and this is a recruitment issue—one of the reasons that people are nervous about going into rural general practice is that they feel that they will be professionally isolated...this may also serve a purpose where the registrar thinks “Well, actually I have got this support. I can do this and I can learn from this now and forever by being part of these consultations and that I will have this to offer for my patients.” So, yes, I think there’s also another benefit that maybe we hadn’t thought about with this.

GP, site 9

### The Reported Clinical Benefits of Using Telehealth Consulting

#### Continuity of Care

Improvements in continuity of care with enhanced shared care for patients afforded better opportunity for triangulated communications between the patient, the GP or nurse, and the specialist, and patient feeling supported in the consultation. This was recognized as important in the learning opportunity for students and doctors-in-training when they had an established relationship with the patient and were then allowed by that patient to be involved in the 3-way consultations between a specialist and the patient and doctor or nurse:

You get to see the GP and you get to see what the specialist does further to that so you kind of get to see a bit more continuity of care and what happens at both levels and that’s pretty valuable learning opportunity

student, site 4

The telehealth consultations gave a unique continuity of care with patients having the consultation in the primary care setting with which they are familiar with someone they know in the room. This aspect of continuity of care appears to give the patients more confidence. This was an often-mentioned theme and was felt to assist patients to “open up” in the consultation if they had an ongoing care-based relationship with the primary care team:

The patients themselves are being heard...that they can come back to the clinic here...they’ve got that one-on-one contact with the doctor visually...I just think that for the patient in particular, it’s giving them confidence in the service that we’re providing here in the clinic.

practice nurse, site 1

The continuity of patient care also enhanced the opportunity for stronger interactions between all those involved in a telehealth consultation. This can additionally lead to the patient feeing supported within the consultation:

They see what the GP is doing with this patient, they can then see the continuity of care with the specialist, they can then see that again when the patient goes back to review that consultation with the doctor on the same day, they’ve got that full flow through and understanding and comprehension of exactly what the total process is with the patient.

practice manager, site 1

I suppose having someone known to them from this practice sit in with them probably they feel more comfortable. Sometimes the nurses do need to interpret a bit about what the specialist is saying...Sometimes they are quite anxious about teleconferencing initially but having the nurse sitting with them helps a lot.

practice nurse, site 1

### Timely Access to Specialists, Quicker Referrals and Follow-Ups, and Access to a Wider Range of Specialists Than Face-to-Face

Telehealth consultations were seen to have the potential to enhance clinical care of patients through allowing access to a range of specialists not limited by geographical proximity:

We can offer patients comprehensive service that’s good for patient care and because we’re rural and we’re away from the city and we don’t have resident specialists in town.

GP, site 4

The patients were actually very impressed at how the telehealth consults went...it’s really massively improved in terms of access to specialists. We probably haven’t used it as much as a rural place...but we had access to specialists that we seriously wouldn’t have had access to otherwise.

GP, site 3

Timeliness was also viewed as an important clinical benefit:

Traditionally with specialists appointments there are waiting lists and there are also the geographical boundaries and the financial restraints...We find with telehealth there is a higher frequency for cancelations to pop up...which allows us to work with the patients availability.

practice manager, site 1

### Normalization of Telehealth Consulting in Clinical Practice

The normalization of telehealth consultations into medical practice systems was facilitated by the perceived benefits and the support provided by the project’s training and team:

Initially I was very skeptical. I couldn’t really see how specialists could get a good perspective on a patient’s health by seeing them over Skype and I actually thought the patients would find it difficult...But I’ve had a complete turnaround...the patients seem to have adjusted to it really well.

nursing manager, site 1

It’s facilitated a process with us thinking about and putting things in place and encouraging the use of the technology to do consults...we’ve got the technology switched on things are in place.

GP, site 4

## Discussion

Our data provide evidence of substantial capacity and potential for telehealth in a range of learning settings and a range of learners, with advantages for both the learners involved and the patient.

It is not always possible to separate completely the educational and clinical benefits of telehealth consultations—they are intricately linked. They afford the opportunity for the student or doctor-in-training to be involved in a unique parallel consulting situation with the patient, the specialist, and the GP or practice nurse. It appears that the benefits are for the patient, the primary care team, and the medical learner, and they coexist and are synergistic.

The success of this form of medical education was apparent in practices that were established in using telehealth as well as practices that have just started using telehealth. A common theme across both types of practices is that they all developed new practices, protocols, and methodologies to enable efficient use of this alternative to face-to-face consultation. Therefore, the process of change was different across the participating practices (and indeed 2 of the participating practices did not make the change to using telehealth consultations for medical education within the time frame of the project).

However, in all those practices where patients consented to “sharing” their telehealth consultation with at least one potential “learner” (either a doctor-in-training or a medical student), the experience proved to be a successful learning experience, provided the consultation was of sufficient length. Additionally, the experience for the patient was deemed to be successful from the clinical perspective. It also allowed the patient to identify their unique role within the learning experience of students or registrars in the general practice linking with specialists, with whom the learner would be unlikely to connect in other circumstances.

### Mental Health: A Unique Opportunity

The unique opportunities enabled by patients with mental health issues who engaged with the learning process are of significant note. Although this is described here as a unique entity, it should be noted that this has the potential to also enhance job readiness in the learner and to have potential impact on future clinical practice. Management of mental health in rural general practice is a key area in which innovation has been sought [14] and telehealth consultations appear to be an ideal solution.

This was a short-term study; practices have changed or modified their use of telehealth consultations to incorporate into it the opportunity for medical education and learning. There has been a positive attitude to change, which has been demonstrated to enhance its efficacy [15]. Some practices were new to telehealth consulting, others experienced, but all those who used telehealth consultations within their practice for medical education report positive outcomes for both patient care and medical education. These positive outcomes were at all levels: patient, learner, practice managers, nurses, and GPs. It appears that having made the change to incorporating telehealth consultations into core business, the process undergoes normalization. Following staff training, practice-specific guidelines, and role allocation, the process is valued and becomes an inherent aspect of patient care.

The benefits of telehealth consulting are patient-centered, allowing a patient to have a consultation with their specialist and GP concurrently, in a new iteration of parallel consulting. However, the benefits do not solely come to the patient. The support in case management is enhanced, the rural practitioner is more connected with peers in a distant location, and there are opportunities for learning at all levels. It enabled conferring with medical colleagues directly and development of joint management plans involving participation of the patient. It also allowed for any misconceptions or lack of understanding to be rectified immediately. This study has demonstrated that there are perceived benefits specifically related to mental health consultations because of the shared experience of being in a consultation with an experienced psychiatrist.

Utilizing telehealth for medical education did require all participating practices to review their policies, practices, and protocols, and in some cases develop these. It is clear that although the principles are the same in each case and all had a designated staff member to organize and facilitate, practices have individual methods of enabling telehealth consultations. There were differences in the equipment used and the room set up with some practices choosing to have facilities in all consulting rooms and others having dedicated rooms. There were also differences with the roles and involvement of practice nurses in the consultation procedure. Another important aspect was that all practices recognized the importance of a “plan B” should there be technical difficulties to enable a simple telephone link up with the specialist.

### Strengths and Limitations

The strengths of this research are in the adherence to the published research protocol, which gave clear direction and scope [6]. Additionally, the research rigor was ensured by adhering as closely as possible to the criteria within the consolidated criteria for reporting qualitative research (COREQ) recommendations.

Factors limiting the study include the short study timeframe and that during the study period there was a change in the financial incentive payments through Medicare.

### Conclusion

The benefits of involving learners in appropriate telehealth consultations can be recognized in terms of clinical benefits for the patient and educational benefits for the learner. Nevertheless, these 2 benefits are not separate entities, but are fused and their relationship is synergistic. Telehealth consulting can also enhance the total care of the patient through the development of professional relationships and shared care between the patients’ GP and a wide range of specialist services.

## Abbreviations

COREQ: Criteria for reporting qualitative research
GP: general practitioners
PGPPP: Prevocational General Practice Placement Program
RRMA: Rural, Remote and Metropolitan Areas
